# Aldosterone induces rapid sodium intake by a nongenomic mechanism in the nucleus tractus solitarius

**DOI:** 10.1038/srep38631

**Published:** 2016-12-09

**Authors:** Hu Qiao, Bo Hu, Hong Zhou, Jianqun Yan, Ru Jia, Bo Lu, Bo Sun, Xiao Luo, Yuanyuan Fan, Nan Wang

**Affiliations:** 1 Key Laboratory of Shaanxi Province for Craniofacial Precision Medicine Research, Xi’an Jiaotong University College of Stomatology, 98# Xiwu Road, Xi’an, Shaanxi 710000, P.R. China; 2 Department of Physiology and Pathophysiology, Xi’an Jiaotong University School of Basic Medical Science, 76# W. Yanta Road, Xi’an, Shaanxi 710061, P.R. China; 3 Department of Orthodontics, Xi’an Jiaotong University College of Stomatology, 98# Xiwu Road, Xi’an, Shaanxi 710000, P.R. China

## Abstract

The purpose of this study was to determine whether aldosterone has a rapid action in the nucleus tractus solitarius (NTS) that increases sodium intake, and to examine whether this effect of aldosterone, if present, is mediated by G protein-coupled estrogen receptor (GPER). Adult male Sprague-Dawley rats with a stainless-steel cannula in the NTS were used. Aldosterone was injected into the NTS at the doses of 1, 5, 10 and 20 ng 0.1 μl^−1^. A rapid dose-related increase of 0.3 M NaCl intake was induced within 30 min and this increase was not suppressed by the mineralocorticoid receptor (MR) antagonist spironolactone (10 ng 0.1 μl^−1^). Water intake was not affected by aldosterone. The GPER agonist G-1 produced a parallel and significant increase in sodium intake, while pre-treatment with GPER antagonist G15 (10 ng 0.1 μl^−1^) blocked the G-1 or aldosterone-induced rapid sodium intake. In addition, sodium intake induced by sodium depletion or low-sodium diet fell within 30 min after injection into the NTS of the MR antagonist spironolactone, while G15 had no effect. Our results confirm previous reports, and support the hypothesis that aldosterone evokes rapid sodium intake through a non-genomic mechanism involving GPER in NTS.

Sodium plays a very important role in the control of extracellular fluid osmolarity and in the maintenance of electrolyte homeostasis^1^. The body sodium balance is maintained by an intricate network of regulatory system that involves the promotion of sodium reabsorption in the kidney and control of sodium intake by the brain^2^.

Aldosterone represents a key factor in the control of this network^3^,^4^ by influencing activity of nucleus tractus solitarius (NTS), which represents the first central synapse for gustatory afferent fibers. The NTS plays an important role in the control of fluid and energy balance in response to signals arising from the periphery^5^,^6^ and lesions of this brain area increase sodium intake^7^. In the NTS, a specialized subpopulation of neurons that express both 11-β-hydroxysteroid dehydrogenase type 2 (HSD2) and MR were identified^8^ and they might be involved in regulation of sodium appetite as they are activated by sodium deficiency^9^,^10^. Recently, Formenti *et al*.^11^ found that chronic infusions of aldosterone into the fourth ventricle increased sodium intake in Wistar Hanover rats in a dose-dependent manner and Koneru *et al*.^12^ support their results. Koneru *et al*. showed that chronic infusions of aldosterone evoked a dramatic increase in sodium intake that was suppressed by shRNA knockdown of mineralocorticoid receptor (MR).

It has become increasingly clear that aldosterone can mediate its actions in cells by controlling transcriptional and translational processes as well as by a faster non-genomic mechanism^13^,^14^. The classical MR is generally responsible for transducing aldosterone-induced genomic signaling effect and also transmits nongenomic actions of aldosterone. However, in the recent years, this mineralocorticoid receptor paradigm has been challenged with the description of effects not affected by MR antagonism^15^,^16^,^17^ and rapid non-genomic aldosterone effects were reported in the MR knockout mouse, suggesting that they might be produced by the involvement of a different receptor^18^. Recently, a growing body of evidence suggests that rapid non-genomic effects of aldosterone are probably mediated via a novel G protein-coupled estrogen receptor (GPER; formerly named GPR30)^17^,^19^,^20^.

GPER, a newly identified receptor, was cloned and described in 1997^21^. It is widely distributed in many tissues, including the placenta, heart, cancer cells, prostate, lymphoid tissue and blood vessels^22^,^23^. Moreover, many immunohistochemical evidences revealed that GPER-immunoreactive cells were present in the NTS^24^,^25^,^26^. Considering the importance of aldosterone in the control of sodium balance and the immunohistochemical evidence such GPER-immunoreactive cells in the NTS may participate in the control of sodium intake, in the present study, we analyzed whether aldosterone had a rapid action in the NTS that it increased sodium intake and examined whether this effect of aldosterone, if present, was mediated by GPER through the use of GPER blockers and activators.

## Results

### Histological analysis to confirm successful injection

**Figure 1:**
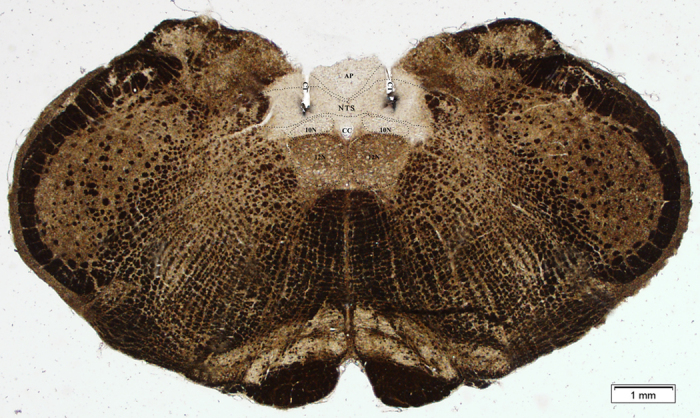
Photomicrograph illustrates bilaterally placed injections in the nucleus of the solitary tract (NTS). Injection sites on each side of the brain stem were indentified by deposits of Pontamine Sky Blue dye. AP, area postrema; CC, central canal; CT, cannula tract; 10N, dorsal motor nucleus of the vagus; 12N, hypoglossal nucleus; scale = 200 μm.

Figure 1 shows the correct cannula placement in the NTS, corresponding to −13.76 to −13.92 mm from bregma according to the placement coordinates described in the atlas of Paxinos and Watson^27^. Most of the injections were localized in the medial portions of the NTS. A total of 237 rats were used in these experiments, and the histological analyses showed that 184 of them had bilateral injections correctly made into the NTS. The data from the 184 rats were used for the following analyses.

Since there was no difference in water or sodium intake between misplaced injection of the drugs and vehicle group, the data from the animals in which the injection sites were not correctly placed within the NTS were not analyzed.

#### Experiment 1. Aldosterone and MR antagonist spironolactone administration in the NTS

**Figure 2:**
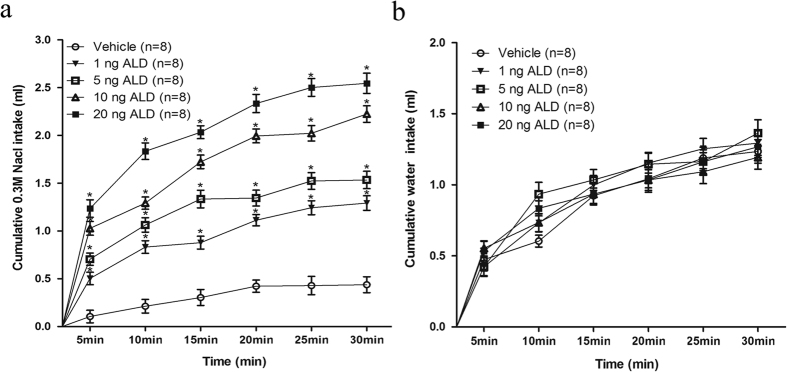
Cumulative intake of 0.3 M NaCl (**a**) and water intake (**b**) by rats that received aldosterone (ALD) injections at different concentrations into the NTS. Error bars show means ± SEM. *P < 0.05, when each treatment group is compared with the vehicle group.

**Figure 3:**
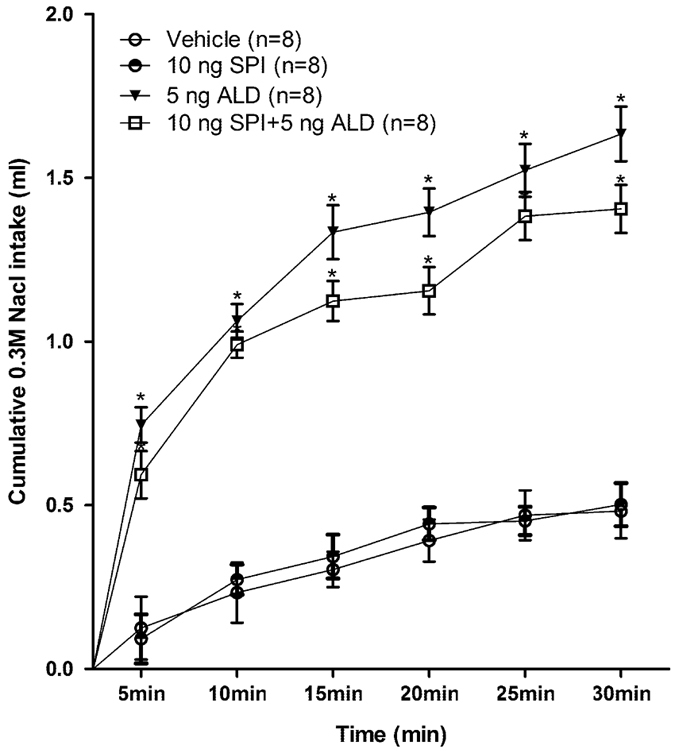
Cumulative 0.3 M NaCl intake by rats that received MR antagonist spironolactone (SPI), or aldosterone (ALD), or spironolactone followed by aldosterone injection into the NTS. Error bars show means ± SEM. *P < 0.05 when each treatment group is compared with the vehicle group.

The microinjections into the NTS of aldosterone at different concentrations, remarkably increased 0.3 M NaCl intake in a dose-dependent manner in all the treated groups compared to the vehicle group (r = 0.953, *P* < 0.05) and the increase occurred rapidly after the drugs were injected, within 15 minutes and continuing to 30 minutes. In order to be concise, these data were shown as the 30-min period (Fig. 2a). In addition, the time course curves were significantly different between treatments [F(4, 35) = 43.68, *P* < 0.05)]. Water intake was not different between the groups [F(4, 35) = 1.07, *P* > 0.05)] (Fig. 2b). The specific MR antagonist spironolactone injected into the NTS 30 min prior to aldosterone injection did not significantly affect the rapid 0.3 M NaCl intake induced by aldosterone (Fig. 3).

#### Experiment 2. Aldosterone, GPER agonist G-1 and antagonist G15 administration in the NTS

**Figure 4:**
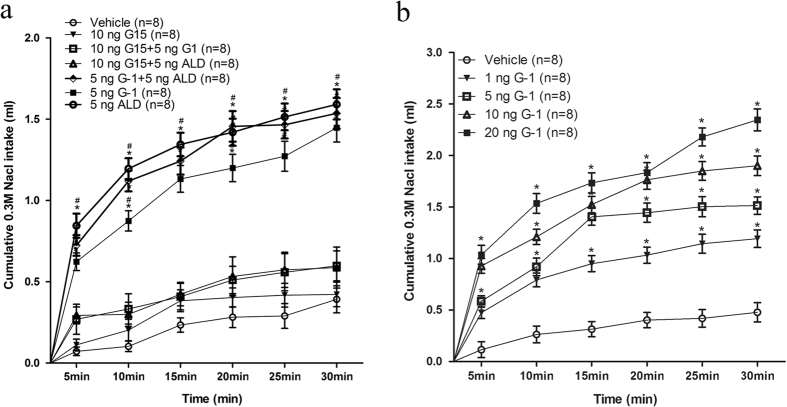
(**a**) Intake of 0.3 M NaCl by rats that received GPER agonist G-1, or vehicle, or aldosterone (ALD), or G-1 + aldosterone (ALD), or GPER antagonist G15 followed by aldosterone (ALD) or G15 followed by G-1 injection. (**b**) Cumulative intake of 0.3 M NaCl by rats that received G-1 injections at different concentrations into the NTS. Error bars show means ± SEM. *P < 0.05 when each treatment group is compared to the vehicle group. ^#^P < 0.05 when each treatment group is compared with the G15 + G-1 group, or to the G15 + aldosterone group.

Figure 4a shows the intake of 0.3 M NaCl during the sodium intake test when the animals received either G-1 or vehicle injections. The GPER agonist G-1 stimulated sodium intake when applied to the NTS. In addition, the intake of sodium stimulated by G-1 was similar to the intake of sodium stimulated by aldosterone (5 ng 0.1 μl^−1^) in both the onset of drinking and the volume consumed [F(3, 28) = 39.56, *P* < 0.05)] (Fig. 4a). Concomitant microinjection of G-1 and aldosterone also stimulated sodium intake, but it was not significantly different from G-1 alone (*P* > 0.05). Pretreatment with the GPER antagonist G15 mostly blocked the sodium intake induced by either aldosterone (*P* < 0.05) or G-1 (*P* < 0.05) (Fig. 4a). And injection of G15 to the NTS had no effect on sodium intake. In addition, the microinjections into the NTS of G-1 at different concentrations also rapidly increased 0.3 M NaCl intake within 30 minutes, similar to the sodium intake stimulated by aldosterone and the time course curves were significantly different between treatments [F(4, 35) = 35.66, *P* < 0.05)].

#### Experiment 3. Low-sodium diet-induced sodium intake by rats treated with injection of MR antagonist or GPER antagonist into the NTS

**Figure 5:**
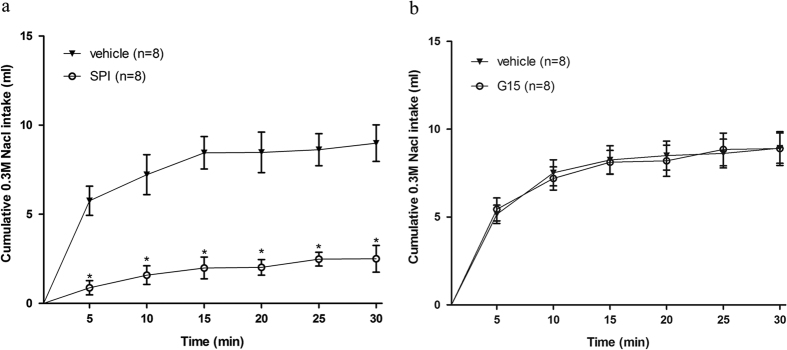
Intake of 0.3 M NaCl induced by 14 days of a low-sodium diet followed by spironolactone (SPI) or G15 treatment. Rats had free access to a low-sodium diet and distilled water for 14 days. Intake of 0.3 M NaCl by rats that received MR antagonist spironolactone (SPI) (**a**) or GPER antagonist G15 (**b**) injection into the NTS after the 2 weeks of low-sodium diet. Error bars show means ± SEM. *P < 0.05 when treatment group is compared with the vehicle group.

Injection of MR antagonist spironolactone into the NTS reduced 0.3 M NaCl intake induced by 14 days of low-sodium diet (2.3 ± 0.9 ml 30 min^−1^ vs. 8.9 ± 2.5 ml 30 min^−1^ in the vehicle group) [F(1, 14) = 42.38, *P* < 0.05] (Fig. 5a). On the other hand, injection of G15 into the NTS produced no change in low-sodium diet-induced intake of 0.3 M NaCl (7.8 ± 2.6 ml 30 min^−1^ vs. 8.5 ± 2.3 ml 30 min^−1^ in the vehicle group) [F(1, 14) = 1.54, *P* > 0.05] (Fig. 5b).

#### Experiment 4. Sodium depletion-induced hypertonic sodium intake by rats treated with a bolus injection of MR antagonist or GPER antagonist into the NTS

**Figure 6:**
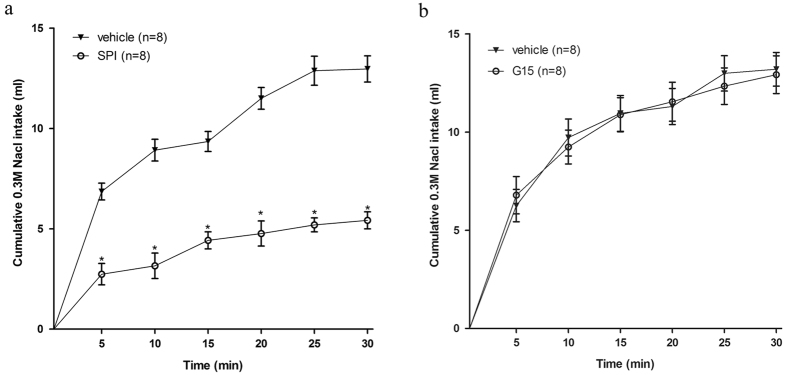
Intake of 0.3 M NaCl induced by 24 h of sodium depletion followed by spironolactone (SPI) or G15 treatment. Rats received an injection of furosemide and next, they had free access to a low-sodium diet for 24 h. Intake of 0.3 M NaCl by rats that received MR antagonist spironolactone (SPI) (**a**) or GPER antagonist G15 (**b**) injection into the NTS after 24 h of low-sodium diet. Error bars show means ± SEM. *P < 0.05 when each treatment group is compared with the vehicle group.

Injection of the MR antagonist spironolactone into the NTS reduced 0.3 M NaCl intake induced by 24 h of sodium depletion due to furosemide treatment followed by 24 h without access to sodium (4.6 ± 0.8 ml 30 min^−1^ vs. 11.3 ± 2.7 ml 30 min^−1^ in the vehicle group) [F(1, 14) = 31.97, *P* < 0.05] (Fig. 6a). On the other hand, injection of G15 into the NTS showed no effect in reducing sodium depletion-induced intake of 0.3 M NaCl (12.8 ± 2.6 ml 30 min^−1^ vs. 14.1 ± 3.3 ml 30 min^−1^ in the vehicle group) [F(1, 14) = 2.07, *P* > 0.05] (Fig. 6b).

## Discussion

The present study shows that the sodium intake can be rapidly raised by application of aldosterone into the NTS. Moreover, the data also demonstrates the importance of GPER in the NTS for the initiation of aldosterone-induced sodium intake. The injection of spironolactone into the NTS reduced sodium intake induced by either low-sodium diet or 24 h of sodium depletion, whereas after injection of G15, the reduction in sodium intake was not observed. Water intake was not affected by these treatments. These results suggest that rapid actions of aldosterone may play some role in the control of sodium intake in the NTS.

The NTS is the important gateway for gustatory and visceral information that make a relay in the parabrachial nucleus (PBN) prior to projecting to the amygdala, thereby forming a major neuraxis for the control of sodium appetite and the taste^28^,^29^,^30^,^31^,^32^. Recent studies showed that 4th V chronic infusions of aldosterone evoked a dramatic increase in sodium intake^11^,^12^. After the infusion, aldosterone would penetrate into the brain parenchyma and the square of the distance increased with the time for drug diffusion^33^,^34^. Therefore, when aldosterone was chronic infused or bolus injected into NTS, the range of diffusion was distinct and different areas might be activated to produce specific sodium intake. In the present study, microinjection of aldosterone into the NTS could promote a rapid and significant increase in the 0.3 M NaCl intake in a dose-dependent manner. This increase in sodium intake was seen as early as 5 minutes after aldosterone injection during the rats’ free access to 0.3 M NaCl. Moreover, this effect was independent of the ability of spironolactone to block the increase in sodium intake stimulated by aldosterone.

Although the injection of aldosterone into the NTS rapidly induced sodium intake, it failed to increase water intake. The behavioral drive to ingest water is controlled by multiple stimuli related to body-fluid hydration, such as hypovolemia, angiotensin II or hyperosmolarity, which promotes thirst and water retention via behavioral and neuroendocrine output centers in the hypothalamus^30^,^35^,^36^. The brain tissue surrounding the rostral third ventricle is also involved in the stimulation of water intake^37^,^38^. Our data are, to some degree, consistent with the studies of Formenti *et al*.^11^ and Koneru *et al*.^12^ who reported that infusion of aldosterone into the 4th V resulted in a little effect on water intake in rats.

The actions of mineralocorticoids in the brain involve both rapid non-genomic mechanisms and slow genomic mechanisms and both are thought to be involved in the control of sodium intake^39^,^40^,^41^. Slow genomic aldosterone action focuses on intracellular MR. In the NTS, a specialized group of aldosterone-sensitive neurons that express HSD2 and MR might represent an important target for aldosterone action in the brain^9^,^10^. Meanwhile, increasing evidence supports the non-genomic actions of aldosterone *in vitro* and *in vivo*^42^,^43^,^44^,^45^,^46^. Notably, Sakai *et al*. found that treatment of rats in the amygdala with aldosterone increased saline intake within 15 minutes after injection and suggested the quick elicitation of behavior might be acting through GABAα/benzodiazepine receptors system rather than MR^47^. Moreover, faster non-genomic aldosterone effects were reported in the MR knockout mouse suggesting that non-genomic aldosterone receptor is clearly distinct from the classical MR^18^. In the present study, the inability of the specific MR antagonist spironolactone to block the effect of aldosterone, along with the rapid elicitation of sodium intake soon after aldosterone treatment, suggests that this local and rapid effect of aldosterone in the NTS may be acting through a novel receptor rather than the classical MR. In addition, a considerable number of studies show that GPER, a G protein-coupled receptor (GPCR) is able to induce rapid responses^17^,^20^, and it is present in NTS^24^,^25^,^26^. Thus, it is possible that the rapid sodium intake we observed, induced by aldosterone, may be triggered by GPER.

G-1 and aldosterone were simultaneously injected into the NTS to further verify whether or not GPER is a common receptor for G-1 and aldosterone. The two agonists did not produce more sodium intake, as compared to G-1 alone, at least partly, supporting GPER as a common pathway for the effects of G-1 and aldosterone.

The involvement of a GPER system was supported by our pharmacological study, which demonstrated that G-1 was equally effective as aldosterone in stimulating a rapid sodium intake. Our results showed that aldosterone and G-1-induced sodium intake was not largely affected by the MR antagonists spironolactone, unlike previous findings in vascular smooth muscle and endothelial cells, where both GPER and MRs contributed to the effects of aldosterone^15^,^19^. However, in the present study, spironolactone lowered the rapid effects of aldosterone on sodium intake slightly, which cues the possibility of the cooperative effect between the membrane associated GPER and MR, and the further possibility that MR might be partly present in the same neurons of GPER. In addition, the effects of G-1 and aldosterone in stimulating a rapid sodium intake were inhibited by pretreating the NTS with the antagonist G15. Similarly, a recent study indicates that in vascular endothelial cells, the rapid vasodilator effects of aldosterone or G-1 were blocked by the GPER antagonist G15^17^. Taken together, these data suggest that the rapid actions of aldosterone in NTS might be mediated, at least in part, through a GPER-dependent pathway.

Another notable observation was that sodium intake induced by either 24 h of sodium depletion or low-sodium diet was reduced by administration of the specific MR antagonist spironolactone, similar to previously reported in another rat strain^11^, whereas the injection of GPER antagonists G15 into the NTS had no effect on sodium intake. These results suggest that sodium intake induced by the administration of furosemide or sodium intake after 14 days of low-sodium diet depends mostly on genomic action of aldosterone.

In conclusion, the present study shows that injection of aldosterone or GPER agonist G-1 into the NTS both induces prompt and significant sodium intake that is suppressed by GPER antagonist G15 but not by spironolactone, whereas sodium intake induced by either chronic low-sodium diet or 24 h of sodium depletion is suppressed by spironolactone not by G15. The results suggest that the rapid actions of aldosterone may be mediated, at least in part, through a GPER-dependent pathway in NTS.

## Methods

### Animals

Adult male Sprague–Dawley rats (provided by Medical Experimental Animal Center of Xi’an Jiaotong University, Shaanxi Province, China) weighing 250 to 300 g were individually housed in a temperature controlled room (24 ± 2 °C) on a 12:12-h light-dark cycle (lights on at 7:00 am) and had ad libitum access to standard diet, distilled water (DW) and 0.3 M NaCl unless otherwise indicated.

### Brain surgery

Rats were anesthetized with an intraperitoneal injection of chloral hydrate (300 mg kg^−1^, i.p.), and secured in a stereotaxic apparatus (SN-2N, Narishige Group, Tokyo, Japan). One stainless steel 23-gauge cannula with two tubes was implanted into the NTS. The stereotaxic coordinates of the NTS were 13.9 mm caudal to bregma, 0.5 mm lateral to the midline, and 7.8 mm below the surface of the skull^27^. The cannula was fixed with three screws and dental acrylic resin, and the obstructor (30 gauge) was inserted. A prophylactic dose of penicillin was administered intramuscularly at the end of surgery. All rats underwent a recovery period of 5 to 7 days before the start of the experiments. The experiments were done in accordance with the Principles of Laboratory Animal Care (NIH Publication No. 85-23) and were approved by the Institutional Animal Care Committee of Xi’an Jiaotong University.

### Microinjection

The drugs were microinjected into the NTS using 1 μl Hamilton syringes (Hamilton, Reno, NV, USA) connected by PE-10 polyethylene tubing to 30-gauge injection cannulas (1 mm longer than the guide cannula). The injection volume into the NTS was 0.1 μl and each injection lasted for 1 min followed by an additional 1 min with the injection cannula left in place; the injection cannula was then replaced with the obturator.

### Drugs

The drugs, such as aldosterone, the selective MR antagonist spironolactone and furosemide were purchased from Sigma-Aldrich (Sigma, St. Louis, MO). The selective GPER agonist G-1 (1-(4-(6-bromobenzo[1,3]dioxol-5-yl)-3a,4,5,9b-tetrahydro-3H-cyclopenta[c]quinolin-8-yl)-ethanone) was purchased from Calbiochem-Novabiochem (San Diego, CA, USA). The highly selective GPER antagonist G15 (4-(6-bromo-benzo[1,3]dioxol-5-yl)-3a,4,5,9b-tetrahydro-3H-cyclopenta[c]quinoline) was obtained from Tocris Bioscience (Ellisville, MO, USA). All the drugs were dissolved in DMSO-H_2_O 1:100 and DMSO-H_2_O 1:100 was used as vehicles. The drugs injected into the NTS were aldosterone and G-1 at the final concentration of 1 ng, 5 ng, 10 ng, 20 ng 0.1 μl^−1^ and spironolactone and G15 10 ng 0.1 μl^−1^. These doses were chosen on the basis of previous studies^11^,^20^,^48^,^49^.

### Behavioral tests

Post-surgery rats were placed in their individual metabolism/feeding-drinking cages, a part of feeding-drinking-activity analyser which measures fluid intake by bottle weight (Cat. No. 41800111213) (UGO Basline Biological Research Apparatus, COMERIO-Varese, ITALY), and underwent training for the two-bottle choice test between water and 0.3 M NaCl. The rats were maintained on standard diet and water, and given daily access to 0.3 M NaCl to drink every day. Once a stable baseline of 0.3 M NaCl intake was achieved (usually after 4 to 5 days), the effects of various drugs were examined. Rats were not fed during the tests.

#### Experiment 1. Aldosterone and MR antagonist spironolactone administration in the NTS

The rats NTS was treated with vehicle or 1, 5, 10, 20 ng 0.1 μl^−1^ aldosterone (*n *=* *8/dose), or vehicle + vehicle (0.1 μl) (*n *=* *8), or the MR antagonist spironolactone (10 ng 0.1 μl^−1^) + vehicle (*n *=* *8), or aldosterone (5 ng 0.1 μl^−1^) + vehicle (*n *=* *8) or with spironolactone (10 ng 0.1 μl^−1^) followed by aldosterone (5 ng 0.1 μl^−1^) after 30 min (*n *=* *8). These rats underwent a two-bottle choice test as explained above (0.3 M NaCl solution versus water) and the cumulative intakes of water and 0.3 M NaCl solution were automatically measured by the feeding-drinking-activity analyser for two hours. The concentration of aldosterone which had a moderate effect on the sodium intake in rats was used in the following experiment 2.

#### Experiment 2. Aldosterone, GPER agonist G-1 and antagonist G15 administration in the NTS

The rats NTS was treated with the GPER agonist G-1 (5 ng 0.1 μl^−1^) + vehicle (*n* = 8), or aldosterone (5 ng 0.1 μl^−1^) + vehicle (*n* = 8), or G-1 (5 ng 0.1 μl^−1^) + aldosterone (5 ng 0.1 μl^−1^) (*n* = 8) or vehicle + vehicle (0.1 μl, *n* = 8), and 0.3 M NaCl solution intake was automatically measured by the feeding-drinking-activity analyser for two hours after the microinjections. To examine the specificity of these effects, the GPER antagonist G15 (10 ng 0.1 μl^−1^, *n* = 8) was administered through the same cannula just prior to the administration of G-1 or aldosterone. In addition, the rats NTS was treated with vehicle or 1, 5, 10, 20 ng 0.1 μl^−1^ G-1 (*n *=* *8/dose), and the cumulative intakes of 0.3 M NaCl solution were automatically measured by the feeding-drinking-activity analyser for two hours.

#### Experiment 3. Low-sodium diet-induced sodium intake by rats treated with injection of MR antagonist or GPER antagonist into the NTS

Rats had free access to low-sodium diet (0.02% NaCl) and distilled water for 14 days. After these two weeks, the rats received an injection of vehicle (0.1 μl, *n* = 8), or spironolactone (10 ng 0.1 μl^−1^, *n* = 8) or G15 (10ng 0.1 μl^−1^, *n* = 8) into the NTS. Two hours after the spironolactone or vehicle injection, or 15 minutes after the G15 injection, access was allowed to the bottles containing water or 0.3 M NaCl. The cumulative intake of 0.3 M NaCl solution was automatically measured by the feeding-drinking-activity analyser for two hours after the microinjections.

#### Experiment 4. Sodium depletion-induced hypertonic sodium intake by rats treated with a bolus injection of MR antagonist or GPER antagonist into the NTS

Rats received a subcutaneous (s.c.) injection of furosemide (20 mg kg^−1^ b.w.). Next, the rats had free access to water and to a low-sodium diet for 24 h. After this 24 h interval, the rats received an injection of vehicle (*n* = 8), or spironolactone (10 ng 0.1 μl^−1^, *n* = 8) or G15 (10 ng 0.1 μl^−1^, *n* = 8) into the NTS. Two hours after the spironolactone or vehicle injection, or 15 minutes after the G15 injection, the rats had free access to the bottles containing water or 0.3 M NaCl. The cumulative intake of 0.3 M NaCl solution was automatically measured by the feeding-drinking-activity analyser for two hours after the microinjections.

### Histology

At the end of the experiments, 2% Pontamine Sky Blue solution (0.2 μl) was injected into the NTS. The rats were then deeply anesthetized with a high dose of chloral hydrate and perfused transcardially with PBS followed by 10% buffered formalin. The brains were removed, fixed, frozen-sectioned (40 μm) in a coronal plane, and analyzed under a light microscope to confirm the injection sites in the NTS according to the atlas of Paxinos and Watson.

### Statistical analysis

Statistical analysis was performed using a Statistical Program for Social Sciences statistical software (SPSS 13.0). All data are presented as means ± standard error of the mean (SEM) and were analyzed using two-way analysis of variance with repeated-measures. Post hoc comparisons were performed using Student-Newman-Keuls multiple comparison test. Linear regression was performed to analyze the correlation between drug dose and effect. *P* < 0.05 was considered statistically significant. Taking into account Fitts’s assumption^50^, sodium and water intake were analyzed as cumulative and non-cumulative data, and since there were no differences in the outcome of the hypothesis testing, such findings were presented as cumulative data as it is more usual.

## Additional Information

**How to cite this article**: Qiao, H. *et al*. Aldosterone induces rapid sodium intake by a nongenomic mechanism in the nucleus tractus solitarius. *Sci. Rep.* **6**, 38631; doi: 10.1038/srep38631 (2016).

**Publisher's note:** Springer Nature remains neutral with regard to jurisdictional claims in published maps and institutional affiliations.
